# Assessing the effect of anesthetic gas mixtures on hyperpolarized ^13^ C pyruvate metabolism in the rat brain

**DOI:** 10.1002/mrm.29274

**Published:** 2022-04-25

**Authors:** Richard Healicon, Catriona H. E. Rooney, Vicky Ball, Ayaka Shinozaki, Jack J. Miller, Sean Smart, Daniel Radford‐Smith, Daniel Anthony, Damian J. Tyler, James T. Grist

**Affiliations:** ^1^ Department of Physiology, Anatomy, and Genetics University of Oxford Oxford United Kingdom; ^2^ Oxford Centre for Clinical Magnetic Resonance Research, Division of Cardiovascular Medicine, Radcliffe Department of Medicine University of Oxford Oxford United Kingdom; ^3^ Clarendon Laboratory, Department of Physics University of Oxford Oxford United Kingdom; ^4^ The PET Centre and The MR Centre, Clinical Medicine Aarhus University and Aarhus University Hospital Aarhus Denmark; ^5^ Nuffield Department of Clinical Neurosciences University of Oxford Oxford United Kingdom; ^6^ Department of Pharmacology University of Oxford Oxford United Kingdom; ^7^ Department of Radiology Oxford University Hospitals Oxford United Kingdom; ^8^ Institute of Cancer and Genomic Sciences University of Birmingham Birmingham United Kingdom

**Keywords:** brain, hyperpolarized, metabolism, MRI

## Abstract

**Purpose:**

To determine the effect of altering anesthetic oxygen protocols on measurements of cerebral perfusion and metabolism in the rodent brain.

**Methods:**

Seven rats were anesthetized and underwent serial MRI scans with hyperpolarized [1–^13^C]pyruvate and perfusion weighted imaging. The anesthetic carrier gas protocol used varied from 100:0% to 90:10% to 60:40% O_2_:N_2_O. Spectra were quantified with AMARES and perfusion imaging was processed using model‐free deconvolution. A 1‐way ANOVA was used to compare results across groups, with pairwise *t* tests performed with correction for multiple comparisons. Spearman's correlation analysis was performed between O_2_% and MR measurements.

**Results:**

There was a significant increase in bicarbonate:total ^13^C carbon and bicarbonate:^13^C pyruvate when moving between 100:0 to 90:10 and 100:0 to 60:40 O_2_:N_2_O % (0.02 ± 0.01 vs. 0.019 ± 0.005 and 0.02 ± 0.01 vs. 0.05 ± 0.02, respectively) and (0.04 ± 0.01 vs. 0.03 ± 0.01 and 0.04 ± 0.01 vs. 0.08 ± 0.02, respectively). There was a significant difference in ^13^C pyruvate time to peak when moving between 100:0 to 90:10 and 100:0 to 60:40 O_2_:N_2_O % (13 ± 2 vs. 10 ± 1 and 13 ± 2 vs. 7.5 ± 0.5 s, respectively) as well as significant differences in cerebral blood flow (CBF) between gas protocols. Significant correlations between bicarbonate:^13^C pyruvate and gas protocol (ρ = −0.47), mean transit time and gas protocol (ρ = 0.41) and ^13^C pyruvate time‐to‐peak and cerebral blood flow (ρ = −0.54) were also observed.

**Conclusions:**

These results demonstrate that the detection and quantification of cerebral metabolism and perfusion is dependent on the oxygen protocol used in the anesthetized rodent brain.

## 1 INTRODUCTION

Hyperpolarized carbon‐13 (^13^C) MRSI via dynamic nuclear polarization is a translational metabolic imaging method with the ability to non‐invasively, non‐destructively, and rapidly measure metabolism in cells, animals, and humans in both health and disease.^1^, ^2^, ^3^, ^4^ Hyperpolarized MRI relies on the transient increase of signal in an isotopically labeled substrate, such as [1–^13^C]pyruvate, to allow the real‐time detection of substrate delivery and its subsequent metabolism into down‐stream metabolites.^5^ Using this approach, a number of studies have demonstrated the ability of hyperpolarized MRI to assess metabolism in the brain, both in animals and humans.^6^, ^7^, ^8^, ^9^, ^10^, ^11^

Key to improving our understanding of many neurological conditions, such as multiple sclerosis and neuromyelitis optica spectrum disorder, are animal models of the disease. This is because clinically acquiring tissue biopsy from the brain, to understand both the natural history of the disease as well as the ongoing pathology before and after therapy, is not routinely possible or ethical. Common to most animal studies is the use of an anesthetic agent, such as isoflurane, aerosolized with oxygen to ensure appropriate immobilization and sedation of the animal. Previous hyperpolarized studies have assessed the use of different anesthetic agents on the measured metabolism of the rodent brain, with changes in lactate and bicarbonate observed depending on the agent and dose used,^12^, ^13^, ^14^ but the effect of the carrier gas composition has not been investigated thoroughly in this context.

Consequently, multiple protocols are often used in pre‐clinical imaging, either with 100% O_2_ ^14^, ^15^, ^16^, ^17^, ^18^, ^19^, ^20^, ^21^, ^22^, ^23^, ^24^, ^25^ or 90:10% O_2_:N_2_O^26^, ^27^, ^28^, ^29^ frequently used. Moreover, not all authors report the carrier gas type and concentration used in their experiments. At first, the rationale for using 100% O_2_ is clear: to compensate for presumed hypoventilation under anesthesia, averting hypoxia. However, in the absence of disease and at rest, oxygen uptake is perfusion‐ rather than diffusion‐limited,^30^ and in rats; it has been shown that the cerebral metabolic rate of oxygen consumption (CMRO_2_) is maintained until very low arterial oxygen tensions (P_a_O_2_).^31^ Therefore, the relative hypoventilation expected in anesthetized animals is unlikely to result in any profound central hypoxia or reduction in cerebral oxidative metabolism.

On the contrary, there is evidence that over‐oxygenation may have detrimental effects on both cerebral perfusion and normal cerebral metabolism. It has been demonstrated that, in rats, cerebral blood flow (CBF) is reduced under hyperoxic conditions,^32^ and conversely that CBF and cerebral blood volume (CBV) are increased by hypoxia.^31^ This effect has been further observed in across species, and with the advent of non‐invasive MR techniques for assessment of CBF and CBV, has been demonstrated in man as well.^33^, ^34^, ^35^ The mechanism by which hyperoxia induces cerebral vasoconstriction has not yet been fully elucidated. It has been demonstrated that raised carbon dioxide tension is a potent vasodilatory stimulus.^36^ One theory proposed is that with increased oxygen tension, oxygen displaces carbon dioxide from hemoglobin binding sites, reducing total blood carbon dioxide content and carbon dioxide‐mediated vasodilation, however this effect is thought to be too small on its own to be responsible for hyperoxic vasoconstriction.^37^ Instead, it has been suggested that hyperoxia disturbs the balance of reactive oxygen and nitrogen species (ROS and RNS). Superoxide anions react with nitric oxide (NO) to produce RNS in a reaction catalyzed by superoxide dismutase 3 (SOD3). Under physiological conditions, NO is a key cerebral vasodilator. With increased superoxide availability under hyperoxic conditions, it is possible that SOD‐catalyzed conversion of NO to ONOO^−^ RNS depletes the pool of NO, and removes tonic NO vasodilation, resulting in cerebral vasoconstriction.^38^ This effect is well‐known clinically, leading to patient‐specific ventilation in the context of perioperative medicine, made feasible by modern ventilators permitting the simultaneous measurement of several relevant parameters across the respiratory cycle. In contrast, most small‐animal pre‐clinical studies do not intubate, and for reasons of space and cost do not measure gas composition. This study aimed to assess the effect of altering oxygen concentration on the detected cerebral metabolism of hyperpolarized [1–^13^C]pyruvate and perfusion of gadolinium in a cohort of rats, to help define the physiology of the healthy brain, illustrating a normal response for comparison with subsequent studies examining pathology.

## 2 METHODS

All animal experiments conformed to (United Kingdom Home Office regulations), to institutional guidelines, and were approved by the United Kigndom Home office Animal Ethics Review Committee.

Seven female Sprague‐Dawley rats were purchased from Charles River (Margate, United Kingdom) at 10 weeks old. Rats were housed together for the duration of the experiments and allowed free access to food and water and were housed on a 12 h/12 h day/night cycle.

Experiments were timed using a laboratory stopwatch, starting when the animal was placed into an anesthetic induction box. Rats were anesthetized with the gas mix used for each individual experiment combined with 2.5% isoflurane. Animals were then weighed and a tail vein cannula was sited. Isoflurane was decreased to 2% when in the 7 T (Agilent Magnet, Santa Clara, USA, Varian Console) scanner. Each rat underwent a total of 3 imaging sessions with the following gas protocols: 100:0%, 90:10%, and 60:40% O_2_:N_2_O with gas ratios equating to a total of 2 L per minute, with a randomized order of scans performed for each animal. Animals were marked with permanent marker over the center of the head for reproducible positioning between scans and transferred to a home built imaging cradle with a 2‐channel ^13^C surface receive coil (Rapid Biomedical, Rimpar, Germany). The oxygen saturations of 3 rats were monitored after removal from the imaging magnet using a paw saturations probe, and averaged over a 2‐min period.

Localizer images were acquired using a ^1^H/^13^C volume transmit/receive coil and a 2D multi‐echo B_0_ mapping sequence was performed with subsequent automated shimming as previously described.^26^

### 2.1 Hyperpolarized ^13^C magnetic resonance spectroscopy and perfusion weighted magnetic resonance imaging

A total of 40 mg [1–^13^C]pyruvic acid (Merck) was mixed with the trityl radical, OX063 (15 mM) (Oxford Instruments, Abingdon, Oxford) and gadolinium (3  μ L, 1:50 dilution in water) (Dotarem) and hyperpolarized for 1 h in a prototype hyperpolarizer system as previously described.^26^ The hyperpolarizer system underwent quality control testing every 4 weeks for the 4 months before experiments. The [1–^13^C]pyruvate signal was monitored with the same coil and pulse sequence at each time point. After 1 h, the hyperpolarized substrate was dissolved using a 4.5 mL mix of sodium hydroxide and 1 mL was injected via the tail vein cannula over 4 s with a 200 mL flush of saline as previously described.^26^

Hyperpolarized spectroscopy was performed using a pulse‐acquire sequence (Gauss excitation pulse, excitation pulse width = 100 ms, receive bandwidth = 5 kHz, Repetition Time (TR) = 1 s, Echo Time (TE) = 0.3 ms, slice thickness = 20 mm, Flip Angle (FA) = 15°, number of time points = 240) with the spectroscopy slice encoding a slab placed to cover the whole brain. Each acquisition was targeted to occur between 20 and 25 min post anesthetic induction.

Animals were transferred while anesthetized to a custom‐built cradle for ^1^H perfusion imaging. A 4‐channel receive proton array (Rapid Biomedical, Rimpar) was placed over the head, locked in place via the ear pins, and the animal was re‐placed in the magnet. A 3D gradient echo‐based sequence was performed (TR = 5 ms, TE = 1 ms, FA = 12°, Radio Frequency (RF) spoiled, acquisition matrix = 256 × 256 × 64, reconstruction matrix = 512 × 512 × 64, FOV = 60 × 60 × 60 mm^3^) for localization.

A single slice dynamic 2D gradient echo acquisition was planned through the mid brain for perfusion imaging as previously described.^39^ Imaging parameters were TR = 20 ms, TE = 10 ms, FA = 20°, acquisition matrix = 128 × 64, FOV = 35 mm, slice thickness = 0.625 mm, total number of time points = 60. 200 μL of gadolinium (Dotarem, Guerbet, France) was pre‐loaded into the tail vein line, and a 200 mL saline flush injected after 2 baseline time points were acquired. Each acquisition was targeted to occur between 40 and 45 min after initial anesthetic induction. Animals were removed from the magnet and allowed to recover for 1 week between each scan.

### 2.2 Image and spectroscopy post‐processing

Perfusion weighted imaging data were post‐processed using model‐free deconvolution^40^ using a region of interest placed in the supplying vessels to provide an arterial input function (AIF) to form CBV (normalized to the AIF), CBF, time to peak (TTP) and mean transit time (MTT) maps. A single region of interest (ROI) was placed within the brain (including the superior sagittal sinus) and mean values for the quantities were calculated.

^13^C spectra from each channel of the 2‐channel receive coil were separately summed in the frequency domain, phased, and combined before being fit in jMRUI v5.2 using pyruvate, lactate, alanine, pyruvate hydrate, and bicarbonate as basis functions. The Cramér–Rao lower bound (CRLB) was calculated for each metabolite. ^13^C Bicarbonate:total ^13^C carbon, bicarbonate:pyruvate, [1‐^13^C]lactate: total ^13^C carbon, [1‐^13^C]lactate:[1‐^13^C]pyruvate and ^13^C bicarbonate: [1‐^13^C]lactate ratios were quantified and the mean for each experiment calculated. The TTP from initial upslope of [1–^13^C]pyruvate was also calculated.

### 2.3 Statistical analysis

Perfusion and metabolic parameters were compared across groups using a 1‐way ANOVA and Bonferroni corrected *t* tests if a significant group effect was observed. The coefficient of variation ($\bar{x}/\sigma$) of the maximum observed solid state signal from the quality control of the hyperpolarizer system was calculated.

Spearman's rank correlation coefficient was computed between all variables, with a Bonferroni correction performed for multiple comparisons. Comparisons were made only once between variables, to reduce the number of correlations performed. *P* < 0.05 was considered statistically significant, with appropriate adjustment of the significance cut off to account for multiple comparisons.

## 3 RESULTS

**FIGURE 1:**
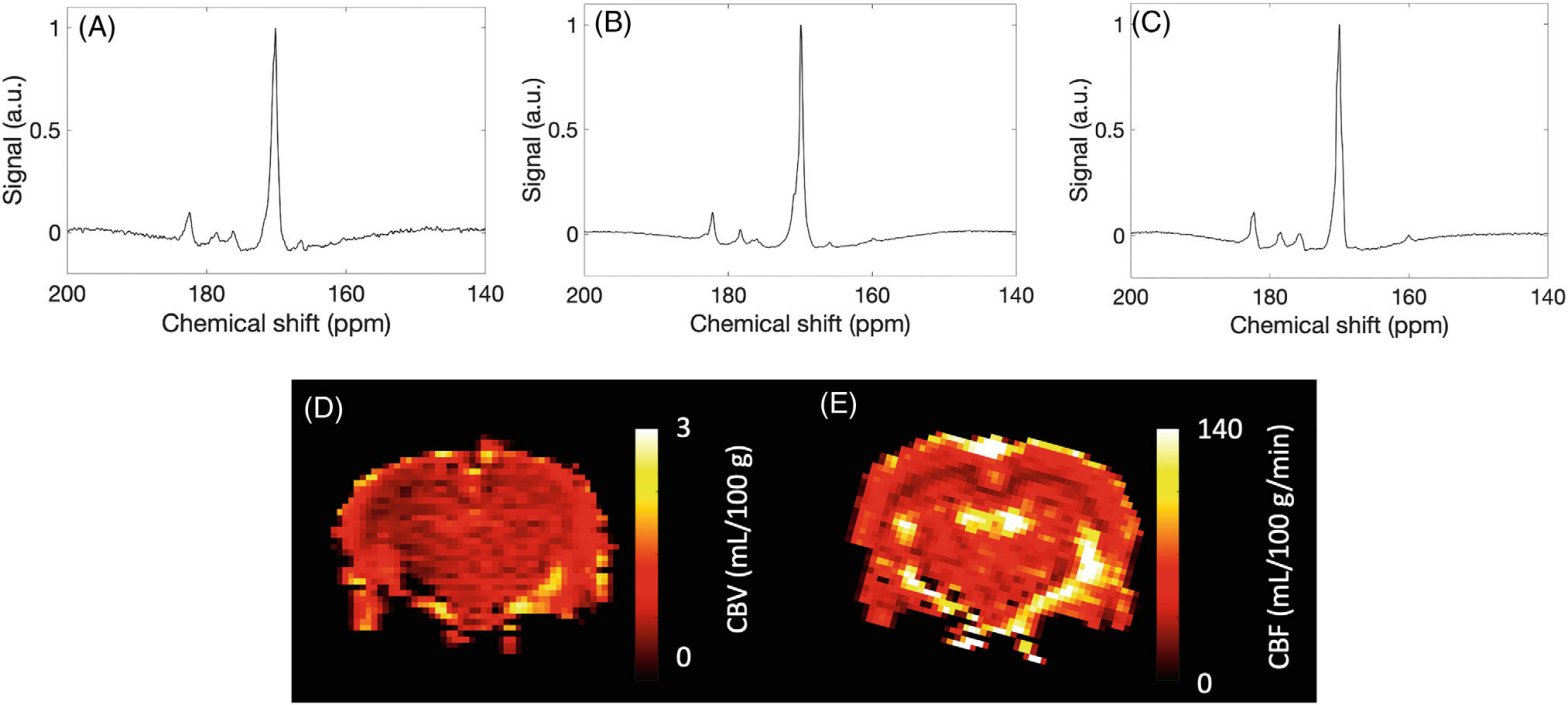
Example summed spectra from the (A) 100:0, (B) 90:10, and (C) 60:40 O_2_:N_2_O% groups, displayed with 5 Hz line broadening and normalized to maximum pyruvate signal. Example (D) CBV and (E) CBF maps from the 60:40 O_2_:N_2_O% group

Hyperpolarized ^13^C spectroscopy and perfusion weighted imaging was successful in all experiments, example summed spectra from 100:0, 90:10%, 60:40% O_2_:N_2_O are shown in Figure 1A‐C, respectively. Example CBV and CBF images from a rat at 60:40% O_2_:N_2_O can also be seen in Figure 1D,E, respectively. Animal weight did not significantly vary over the course of the experiments (*P* = 0.86), and the coefficient of variation of the solid state build up signal from [1–^13^C]pyruvate over 3 months was 7%. Oxygen saturations for animals were consistently between 95‐100% after imaging.

### 3.1 Bicarbonate based metabolic measures significantly vary with gas protocol

**FIGURE 2:**
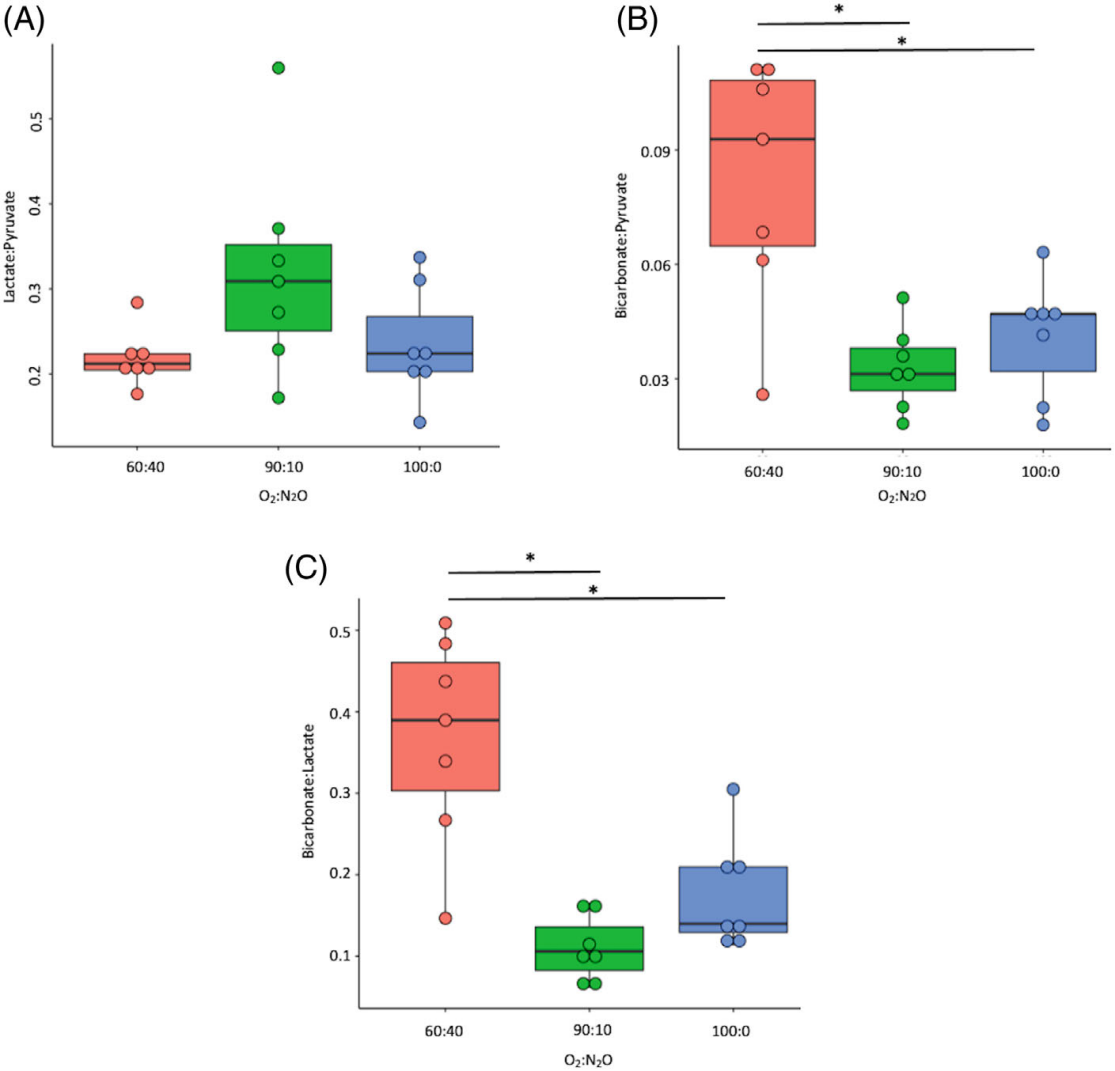
Lactate:^13^C pyruvate (A), bicarbonate:^13^C pyruvate (B), and bicarbonate:lactate (C) results from all groups. *P < 0.05 after correction for multiple comparisons

**FIGURE 3:**
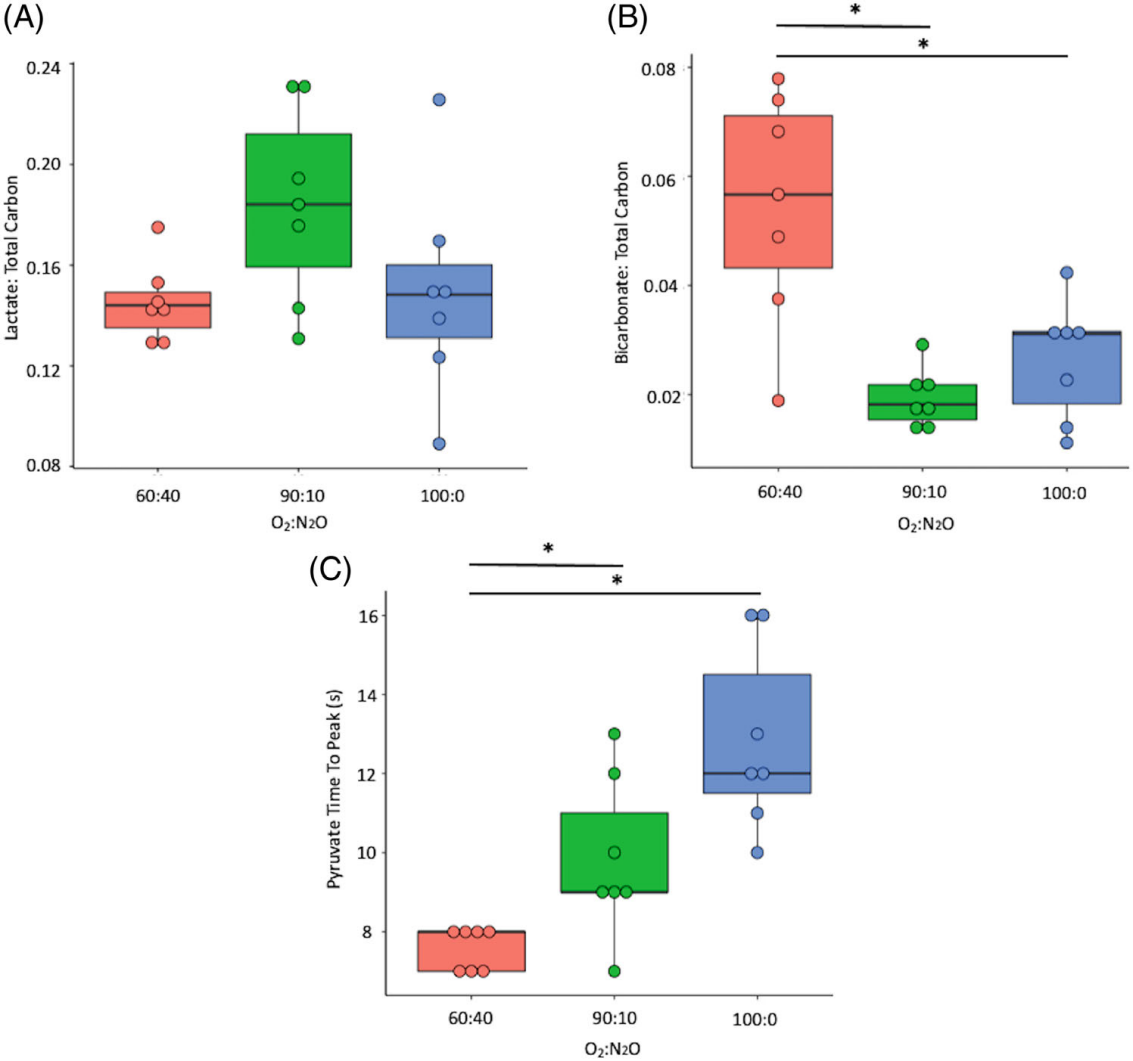
[1‐^13^C]Lactate:total ^13^C carbon (A), ^13^C bicarbonate:total ^13^C carbon (B), [1‐^13^C]pyruvate time to peak results from all groups, *P < 0.05 after correction for multiple comparisons

The ^13^C lactate: ^13^C total carbon or ^13^C lactate:^13^C pyruvate did not significantly change between gas conditions (*P* = 0.09 and *P* = 0.07, respectively) with results shown in Figures 2A and 3A. However, ^13^C bicarbonate:[1‐^13^C]pyruvate and ^13^C bicarbonate:total ^13^C carbon ratio results shown in Figures 2B and 3B, were significantly elevated and higher in the 60:40% O_2_:N_2_O in comparison to the 100:0% O_2_ (0.08 ± 0.03 vs. 0.04 ± 0.01, *P* = 0.0009 and 0.05 ± 0.02 vs. 0.03 ± 0.01, *P* = 0.005, respectively) and between 60:40% and the 90:10% O_2_:N_2_O groups (0.08 ± 0.03 vs. 0.03 ± 0.01, *P* = 0.001 and 0.05 ± 0.02 vs. 0.019 ± 0.005, *P* = 0.0005), but did not significantly differ between 100:0% and 90:10% O_2_:N_2_O groups (*P* = 0.77).

The ^13^C bicarbonate:[1‐^13^C]lactate ratio was significantly different between the 100:0% and 60:40% O_2_:N_2_O (0.18 ± 0.06 vs. 0.4 ± 0.1, respectively, *P* = 0.001) and between 90:10% and 60:40% O_2_:N_2_O (0.11 ± 0.04 vs. 0.4 ± 0.1, respectively, *P* = 0.11), but not between the 100:0% and 90:10% O_2_:N_2_O groups (*P* = 0.34). [1‐^13^C]pyruvate TTP significantly varied between 60:40% and 100:0% O_2_:N_2_O (7.5 ± 0.5 s vs. 13 ± 2 s, respectively, *P* = 0.0001), and between 60:40% and 90:10% O_2_:N_2_O (7.5 ± 0.5 s vs. 10 ± 2 s, respectively, *P* = 0.01), but not between 90:10% and 100:0% O_2_:N_2_O (*P* = 0.07), see Figure 3C.

[1‐^13^C]pyruvate, [1‐^13^C]lactate, and ^13^C bicarbonate Cramer–Rao lower bound estimates were not significantly different over the gas groups (pyruvate, 3 ± 2%, 11 ± 6%, 0.8 ± 0.5%, *P* = 0.09, lactate, 14 ± 7%, 11 ± 6%, 3 ± 2%, *P* = 0.08, bicarbonate, 181 ± 181%, 104 ± 55%, 14 ± 11%, *P* = 0.17, 100:0, 90:10, 60:40 O_2_:N_2_O%, respectively).

### 3.2 Perfusion measures are less sensitive to oxygenation protocol

**FIGURE 4:**
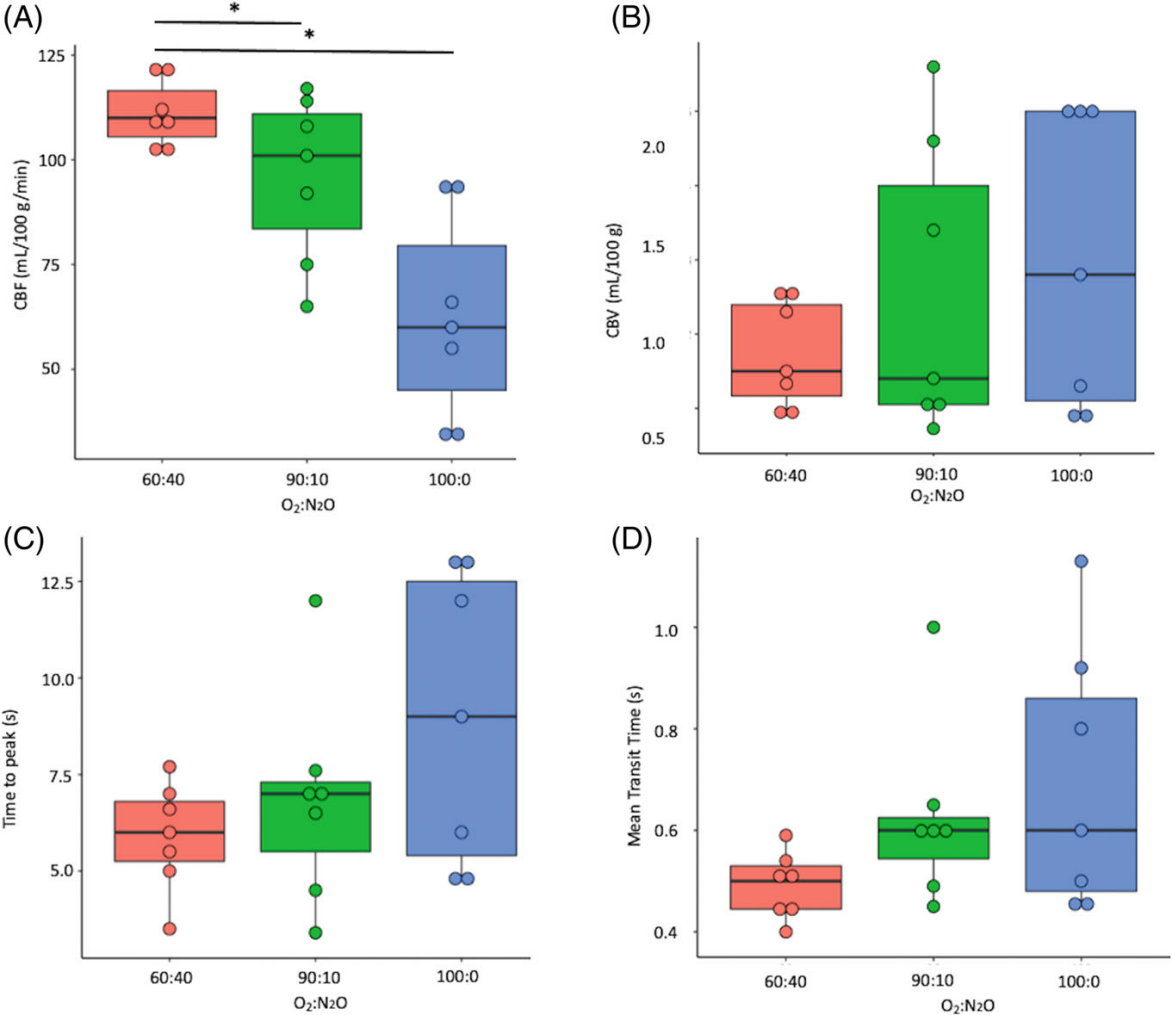
Cerebral blood flow (CBF) (A), cerebral blood volume (CBV) (B), time to peak (TTP) (C) results and mean transit time (MTT) (D) results from all groups. *P < 0.05 after correction for multiple comparisons

**FIGURE 5:**
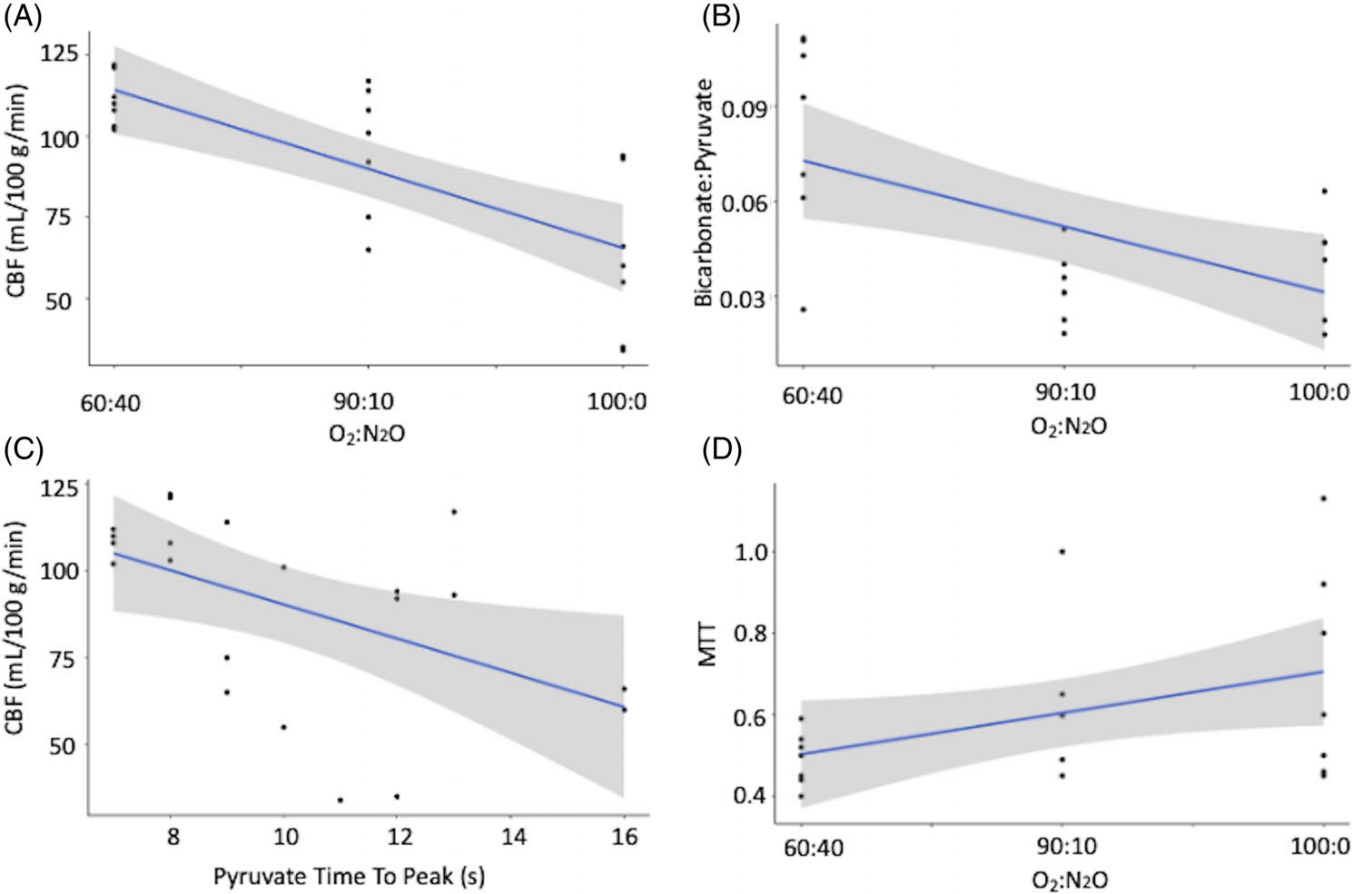
Significant correlation results between CBF and gas protocol (A), ^13^Cbicarbonate: [1‐^13^C]pyruvate (B) [1‐^13^C]pyruvate time to peak with CBF (C) and mean transit time (MTT) with gas protocol (D)

There was significantly elevated CBF as N_2_O was increased and O_2_ decreased, with differences between 60:40 and 100:0 O_2_:N_2_O% (111 ± 7 vs. 62 ± 23 mL/100 g/min, respectively, *P* = 0.003), and between 100:0 and 90:10 O_2_:N_2_O% (62 ± 23 vs. 96 ± 18 mL/100 g/min, respectively, *P* = 0.003), but not between 60:40 and 90:10 O_2_:N_2_O% (*P* = 0.31). Results for CBV, TTP, and MTT were not significant (shown in Figure 4B‐D, respectively, all *P* > 0.05). There were significant correlations between CBF and gas protocol (ρ = −0.75, *P* < 0.001), bicarbonate:^13^C pyruvate and gas protocol (*r* = −0.47, *P* = 0.03), between CBF and ^13^C pyruvate TTP (ρ = −0.54, *P* = 0.001), and between MTT and gas protocol (ρ = 0.41, *P* = 0.06), see Figure 5A‐D, respectively. There was a significant correlation between CBF and bicarbonate:total ^13^C carbon (ρ = 0.42, *P* = 0.05) but not bicarbonate:^13^C pyruvate (ρ = 0.37, *P* = 0.09), and significant correlations between increasing CBF and pyruvate (ρ = 0.48, *P* = 0.02), lactate (ρ = 0.57, *P* = 0.007), and bicarbonate (ρ = 0.56, *P* = 0.008) CRLB.

## 4 DISCUSSION

This study demonstrated the effect of changing both oxygen and NO on the measurements of both cerebral metabolism and perfusion in the anesthetized rodent brain. Here, it was clearly demonstrated, using the same animals, that it is possible to modulate apparent pyruvate dehydrogenase (PDH) activity—as heralded by the change in bicarbonate signal as the animals were moved from 100:0% O_2_ to 60:40% O_2_:N_2_O. Although the exact cellular origin of the bicarbonate signal is still contested, and we cannot directly probe astrocytes, neurons, pericytes, microglia, and oligodendrocytes separately in the same animal, it may be because of the increase in neuronal metabolism because of elevated blood flow and availability of lactate from the neuron‐astrocyte lactate shuttle.^41^ These results demonstrate that any changes in ^13^C bicarbonate production, as assessed by using the ^13^C bicarbonate:[1‐^13^C]lactate (a measure of TCA:glycolytic metabolism occurring within the brain) ^13^C bicarbonate:[1‐^13^C]pyruvate, and ^13^C bicarboante:total ^13^C carbon ratios (measures of the total metabolism of pyruvate by PDH), measured at higher O_2_ concentrations in experiments may be confounded by reduced cerebral perfusion, and therefore, have a much higher statistical uncertainty, as demonstrated by the correlation between metabolite CRLB and increasing CBF in this study. Although these results do show the benefit of adding in N_2_O and decreasing O_2_ to recover cerebral oxidative metabolism, they are by no means exhaustive and further optimisation work should be performed. Interestingly, the bicarbonate:lactate ratio observed in the 60:40% protocol was the closest to that observed in the awake healthy human brain (0.4 ± 0.1 vs. 0.32 ± 0.15, respectively)^2^ and this may point to the protocol providing more physiological results than at 100% O_2_. Indeed, N_2_O has been show to recover perfusion and CMRO_2_ in a previous study, where isoflurane commonly depresses cerebral metabolism—this study has expanded on this initial result by demonstrating this using both perfusion and metabolic measurements in a non‐invasive manner over a range of gas carrier protocols.^42^ A recent study has investigated metabolism in the awake rodent brain, showing changes in ^13^C derived results across different aesthetic regimes.^14^ However, an awake rodent study has other confounders (e.g., the added stress response because of gradient noise and heating and the injection of pyruvate while awake). In essence, this study furthers the challenge faced in interpreting and translating pre‐clinical studies, with data potentially confounded by many factors including anesthetics, the use of pulse sequences that are not realistic for translation to clinical systems either because of gradient slew rates that would induce peripheral nerve stimulation in a patient, high specific absorption rate radio frequency pulses that would not be allowed because of patient safety restrictions, and the ability of a disease model to fully replicate the human condition.

Of note, it was not possible to determine the oxygen protocol used in some previously published studies. This may be a point for future consideration in the reporting of studies to enable good reproducibility and open science.

### 4.1 Limitations

First, this work did not include a medical air protocol—used by a small number of groups primarily in the United Kingdom and Europe—this was because this would have required alterations to the anesthetic induction, requiring a much higher initial dose of isoflurane, used for the other gas protocol. Assessing the metabolism of [1–^13^C]pyruvate with a medical air protocol may provide further insights into the changes associated with oxygen at lower concentrations than used here. Furthermore, these results do not represent the normal human condition because they use isoflurane for anesthesia. The volumes of pyruvate injected in other studies varied from this study (commonly over 1 mL), which we are not able to reproduce because of license restrictions in our institution, and this may also provide a source of discrepancy between these results and others previously published. Investigation into changes in arterial blood gases and pressure may elucidate further mechanisms behind this study; however, this was not possible on our animal license. Further study analysis could include the use of kinetic modeling to understand the change in apparent metabolic rate constants, however, approaches sensitive to low signal‐to‐noise ratios of each individual spectroscopic time point, and so were not used here.^43^ However, it is known that rate constants correlate with the area under the curve approached used in this study.^44^

Furthermore, the absolute liquid state polarization of the [1–^13^C]pyruvate was not measured in this study; however, the 7% coefficient of variation in the solid state signal, which correlates with the liquid state signal, suggests that this was not a significant factor in detected metabolism in the study. Finally, the impacts of other anesthetic agents such as propofol or sevoflurane were not investigated in this study, and the work could be expanded to include this in future.

## 5 CONCLUSION

In conclusion, this study has assessed the impact of different oxygen protocols on the measured metabolism of [1–^13^C]pyruvate in the anesthetized rodent brain, revealing differences in apparent bicarbonate production with increasing levels of N_2_O. This will be of use in future studies of metabolism in the rodent brain with hyperpolarized MRI.
